# Modulation of soluble receptor for advanced glycation end-products (RAGE) isoforms and their ligands in healthy aging

**DOI:** 10.18632/aging.101860

**Published:** 2019-03-23

**Authors:** Francesco Scavello, Filippo Zeni, Calogero C. Tedesco, Emanuela Mensà, Fabrizio Veglia, Antonio Domenico Procopio, Anna Rita Bonfigli, Fabiola Olivieri, Angela Raucci

**Affiliations:** ^1^Unit of Experimental Cardio-Oncology and Cardiovascular Aging, Centro Cardiologico Monzino-IRCCS, Milan, Italy; ^2^Unit of Biostatistics, Centro Cardiologico Monzino-IRCCS, Milan, Italy; 3 Department of Clinical and Molecular Sciences, DISCLIMO, Università Politecnica delle Marche, Ancona, Italy; 4 Center of Clinical Pathology and Innovative Therapy, IRCCS INRCA, Ancona, Italy; 5 Scientific Direction, IRCCS INRCA, Ancona, Italy; * Equal contribution

**Keywords:** RAGE isoforms, aging, inflammaging, biomarker, cardiovascular risk, obesity

## Abstract

The receptor for advanced glycation end-products (RAGE) recognizes several ligands involved in inflammatory diseases. Two circulating soluble isoforms exist: esRAGE derived from alternative splicing and cRAGE generated by the membrane-bound RAGE (FL-RAGE) proteolysis. Together, esRAGE and cRAGE constitute sRAGE and function as decoy receptors preventing FL-RAGE/ligands binding.

We determined serum concentration of both, esRAGE and cRAGE, and their ligands AGEs, HMGB1 and S100A8/A9 in a healthy population of 169 subjects aged 20-90 years. cRAGE showed a negative (r=-0.375, P<0.0001) while AGEs (r=0.160, P=0.0384) and S100A8/A9 (r=0.207, P=0.0091) a positive correlation with age. esRAGE did not change during aging and inversely correlated with Hemoglobin, ALT, insulin, HOMA index, Waist-Hip ratio (W/H), Waist Circumference (WC) and positively with AGEs. cRAGE exhibited also an inverse correlation with WC, W/H, PAI-1, HMGB1, AGEs and S100A8/A9. Age, W/H, HMGB1, S100A8/A9 and AGEs are independent predictors of cRAGE, whereas W/H and AGEs associate with esRAGE. Treatment of cells with glycated albumin reduced cRAGE production and upregulated FL-RAGE.

These results indicate that in a healthy population cRAGE is a biomarker of aging while esRAGE represents a more reliable marker of obesity and insulin resistance. Hence, sRAGE isoforms levels could be differentially associated with age-related diseases risk factors.

## Introduction

The receptor for advanced glycation end-products (RAGE) belongs to the family of pattern recognition receptor (PRR) that recognizes several ligands, such as advanced glycation end-products (AGE), some S100/calgranulins, amyloid-beta peptide, High Mobility Group Box 1 protein (HMGB1) and extracellular matrix proteins [1,2].

Structurally, the membrane-bound full-length form of RAGE (FL-RAGE) is composed by three extracellular Ig-like domains, a single transmembrane helix and a short intracellular domain [1]. Binding of RAGE to its ligands activates several pathways including MAPKs and NF-κB, thus modulating all steps of the inflammatory process, such as cell migration, adhesion and proinflammatory molecules production [3–7]. In physiological conditions, RAGE is predominantly expressed in the lung compared to other tissues [8–11]; however, the rise of its levels is induced by ligands accumulation at the injured sites and is associated with the onset and progression of several chronic inflammatory diseases [12–15].

The RAGE primary transcript undergoes alternative splicing producing both species- and tissues-specific coding and non-coding variants [16–19]. In human, the variant hRAGE, encoding for FL-RAGE, is the most abundant RAGE isoform [19,20]. The variant hRAGE_v1 encodes for the secreted esRAGE, which is characterized by a unique C-terminal 9 amino acids sequence, and represents the main if not the only detectable circulating soluble protein generated by alternative splicing, since all the other soluble variants are predicted to be targeted to nonsense-mediated decay (NMD) without producing the corresponding protein products [20–22]. A soluble receptor, denominated cleaved RAGE (cRAGE), can also derive from the proteolysis of FL-RAGE ectodomain by the proteases ADAM10 and MMPs [23–26]. Both, esRAGE and cRAGE, are collectively named sRAGE and, in most of the cases, function as endogenous protective decoy receptors binding ligands with affinity similar to FL-RAGE and preventing activation of its signaling [8,14,24,27,28].

Cross-sectional studies on human population have suggested circulating sRAGE as potential biomarker of several pathologies although with conflicting data regarding diabetes and cardiovascular diseases [29–32]. Decreased levels of circulating sRAGE or esRAGE have been reported in patients with hypertension [33], metabolic syndrome (MS) [34], in the early phase of type 2 diabetes [35,36] and obesity suggesting sRAGE as an early predictor of cardiovascular risk. Interestingly, sRAGE levels seems to be related to body composition. Lower total sRAGE levels associate with increased body mass index (BMI), waist circumference (WC) and fat mass [37,38]. Recently, Prakash et al. showed that levels of sRAGE decline with age and inversely associate with BMI and fat free mass (FFM) in an apparently healthy population [39]. Healthy centenarians have a significant higher plasma sRAGE amount than healthy young subject [40]. These findings put forward that sRAGE could be a marker of healthy aging and longevity. Nevertheless, none of these studies quantified specifically esRAGE or cRAGE levels. It is possible that aging influences differently the mechanisms of sRAGE isoforms generation; therefore, determining simultaneously circulating esRAGE and cRAGE could be useful to discriminate their specific contribution to sRAGE decline and association to age-related disease risk factors.

In the present study, we simultaneously determined both sRAGE isoforms, esRAGE and cRAGE, and some of their ligands such as AGEs, HMGB1 and S100A8/A9 in a cohort of healthy subjects ranging from 20- to 90- years, with the aim to disentangle their age-related trends.

## RESULTS

### Population features

Table 1 shows features of 169 healthy subjects aged 20-90 yrs stratified into three age groups according to World Health Organization (WHO [41,42];): young (≤45 yrs), middle age (46-64 yrs) and elderly-old (≥65 yrs). Young subjects (21.3%) were less represented than middle age (42.6%) and older (36.1%) individuals. The elderly-old cohort was taking more medications than the middle age and younger cohorts (73.8% vs 37.5% and 22.2%, respectively). Anthropometric measurements such as WC and W/H, lipid (Triglycerides, Cholesterol, LDL), liver (AST) and iron (Transferrin) metabolism, glycometabolic indicators (Glucose, HbA1c), Azotemia, inflammation markers (CRP, IL6), number of hypertensive subjects and medications (Antihypertensive, Antiplatelet, Gastro protectant, Micronutrients, Lipid-lowering) increased, while number of white blood cells (WBC) decreased across the age groups (Table 1). Correlation analysis between variables are reported in Supplementary Table 1.

**Table 1:** Biochemical, metabolic and anthropometric variables and medication therapy of healthy subjects divided in three age groups.

**Variables**	**≤45 yrs**	**46-64 yrs**	**≥65 yrs**	**P trend**
**n**	**36**	**72**	**61**	
**Sex (n/% male)**	17/46	35/48	24/39	0.3803
**BMI (Kg/m^2^)**	25.31 ± 4.30	27.17 ± 4.31	27.05 ± 3.31	0.0700
**WC (cm)**	84.89 ± 12.93	91.10 ± 11.32	93.95 ± 8.65	**0.0002**
**H (cm)**	99.71 ± 9.87	103.22 ± 8.66	103.39 ± 7.84	0.0700
**W/H**	0.85 ± 0.09	0.88 ± 0.08	0.91 ± 0.06	**0.0006**
**WBC (10^3^/uL)**	6.89 ± 1.57	6.38 ± 1.70	6.18 ± 1.63	**0.0495**
**RBC (10^6^/uL)**	4.85 ± 0.45	4.62 ± 0.40	4.67 ± 0.39	0.0900
**Triglycerides (mg/dL)**	73.00 (53.00-118.0)	83.50 (55.00-112.50)	97.00 (80.00-142.00)	**0.0600**
**Cholesterol (mg/dL)**	197.31 ± 35.69	215.94 ± 40.89	218.51 ± 41.89	**0.0215**
**HDL (mg/dL)**	54.28 ± 12.93	59.92 ± 15.36	56.33 ± 15.33	0.7500
**LDL (mg/dL)**	113.49 ± 30.79	127.25 ± 37.60	132.31 ± 33.35	**0.0152**
**ApoAI (mg/dL)**	171.51 ± 31.16	181.18 ± 34.89	175.33 ± 31.70	0.7800
**ApoB (mg/dL)**	100.26 ± 38.96	104.89 ± 29.03	107.16 ± 32.87	0.3300
**Insulin (uiU/mL)**	4.97 (3.85-7.00)	4.56 (3.18-6.16)	5.34 (3.60-7.15)	0.9000
**Glucose (mg/dL)**	89.64 ± 8.54	96.11 ± 10.70	94.16 ± 9.46	0.0900
**HOMA Index**	1.06 (0.89-1.52)	1.02 (0.74-1.51)	1.15 (0.78-1.75)	0.6000
**HGB (g/dL)**	14.17 ± 1.15	14.23 ± 1.20	13.93 ± 1.09	0.2400
**HbA1c (%)**	5.45 ± 0.36	5.77 ± 0.43	5.75 ± 0.41	**0.0027**
**GGT (U/L)**	44.00 ± 14.98	45.21 ± 16.93	49.44 ± 13.57	0.0700
**AST (U/L)**	19.00 (16.00-24.00)	19.00 (15.00-22.50)	23.00 (19.50-27.00)	**0.0019**
**ALT (U/L)**	35.00 (30.00-41.00)	37.00 (32.00-41.50)	35.00 (32.00-42.00)	0.5700
**Transferrin (mg/dL)**	287.11 ± 39.56	247.49 ± 39.89	248.79 ± 45.46	**0.0003**
**Ferritin (ng/mL)**	41.6 (16.3-100.70)	99.50 (54.10-155.70)	69.80 (44.00-131.10)	0.1200
**Azotemia (mg/dL)**	31.00 (27.00-37.00)	37.00 (33.00-43.50)	39.00 (34.00-48.00)	**<0.0001**
**Creatinine (mg/dL)**	0.81 ± 0.20	0.83 ± 0.18	0.88 ± 0.25	0.1000
**PAI-1 (ng/mL)**	17.62 (12.39-24.33)	17.26 (13.25-25.66)	19.61 (13.36-30.05)	0.0900
**CRP (mg/L)**	1.23 (0.63-2.22)	1.26 (0.66-3.17)	2.17 (1.15-5.33)	**0.0307**
**IL6 (pg/mL)**	1.16 (0.82-1.64)	1.37 (0.97-2.38)	2.39 (1.71-3.52)	**<0.0001**
**Smoking (n/%)**	10/27	19/26	9/14	0.1018
**Hypertension (n/%)**	4/10	15/20	34/55	**<0.0001**
**Antihypertensive (n/%)**	2/5.6	14/19.4	32/52.5	**<0.0001**
**Antiplatelet (n/%)**	0/0	3/4.2	8/13.1	**0.0070**
**Gastro protectant (n/%)**	1/2.8	4/5.6	8/13.1	**0.0490**
**Micronutrients (n/%)**	0/0	0/0	5/8.2	**0.0090**
**CNS depressants (n/%)**	2/5.6	3/4.2	8/13.1	0.1000
**Thyroid therapy (n/%)**	0/0	2/2.8	3/4.9	0.2000
**Lipid-lowering (n/%)**	0/0	4/5.6	10/16.4	**0.0030**

Variables with Gaussian distribution are expressed as mean ± SD; variables with skewed distribution as median and interquartile range. In bold statistically significant P trend value. BMI=body mass index; WC=Waist Circumference; H=Hip; W/H=Waist/Hip ratio; WBC=White blood cells, RBC=Red blood cells; HDL=High Density Lipoprotein; LDL=Low Density Lipoprotein; ApoAI=Apolipoprotein AI; ApoB=Apolipoprotein B; HGB=Hemoglobin; HbA1c=glycated hemoglobin; GGT=Gamma Glutamyl transferase; AST=Aspartate transaminase; ALT=Alanine transferase; PAI*‐*1=Plasminogen activator inhibitor‐1; CRP= C-reactive Protein; IL6=Interleukin 6; CNS=Central Nervous System.

### cRAGE but not esRAGE decreases with aging

**Figure 1:**
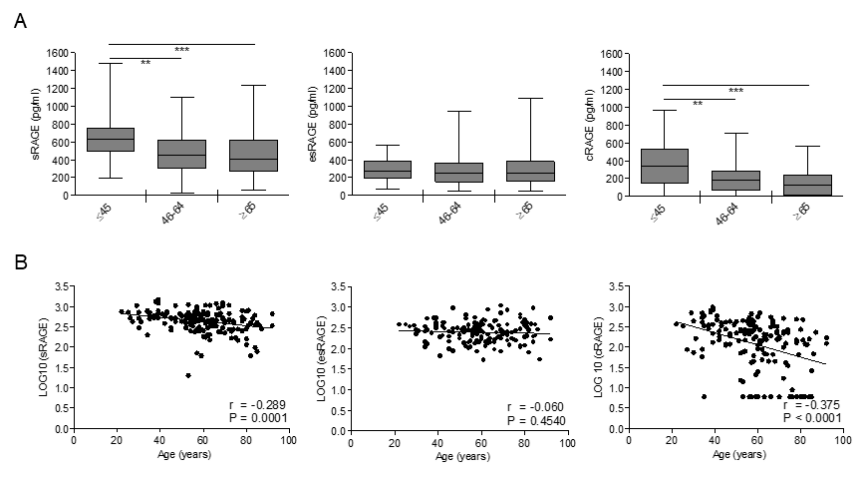
**Circulating cRAGE but not esRAGE decreases with aging in healthy subjects.** cRAGE values were derived by subtracting esRAGE values from the total sRAGE. (**A**) Bars represent serum levels of total sRAGE, cRAGE and esRAGE in three age groups of a healthy population, young (≤45 yrs; n=35-37), middle age (46-64 yrs; n=66-72) and elderly-old (≥65 yrs; n=59-60). Data are in the original units. Values are expressed as median and interquartile range. **P < 0.01; ***P < 0.0001; Kruskal-Wallis with Dunn’s multiple comparison test. (**B**) Scatter plots showing correlation between age and sRAGE, cRAGE or esRAGE. Variables showed a skew-ness distribution and were log-transformed. r=Pearson’s coefficient. P<0.05 was considered statistically significant; n=159-169.

In order to determine the influence of age on circulating RAGE isoforms, we measured total sRAGE, esRAGE and cRAGE in the serum subjects of our cohort. Total sRAGE and cRAGE levels displayed a progressive decrease across the age groups with a significant difference between the young and middle age or elderly-old groups (Fig. 1A). sRAGE and cRAGE variations showed a significant inverse correlation during aging that is stronger for cRAGE (Fig. 1B). In contrast, levels of esRAGE remained almost constant among the groups and showed no correlation with age (Fig. 1). Distributions of all RAGE isoforms were not different between subjects affected and not affected by hypertension, therefore, hypertensive individuals were not removed from the studied population (log_sRAGE P=0.695; log_esRAGE P=0.658; log_cRAGE P=0.159).

**Figure 2:**
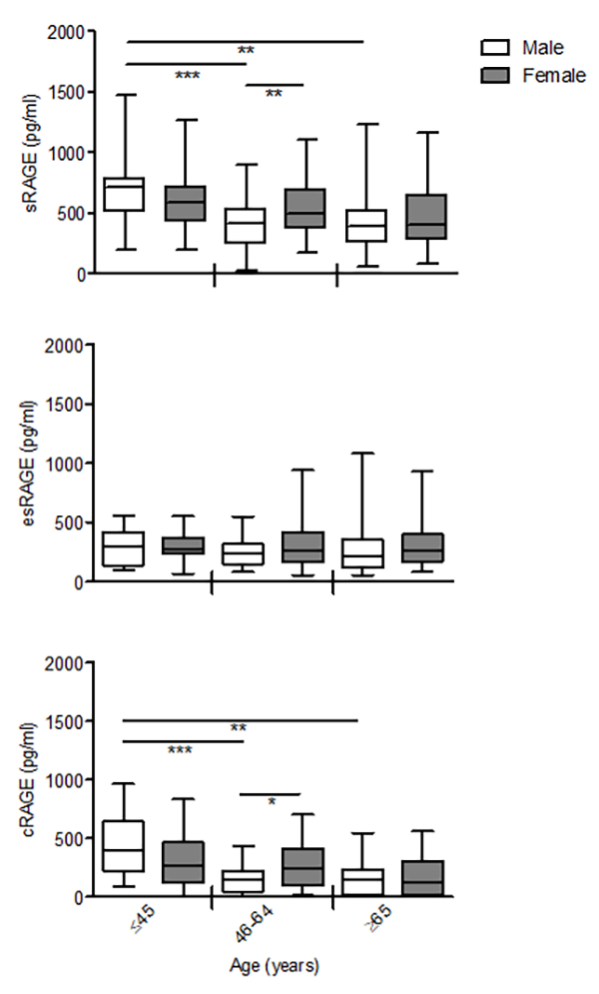
**The effect of age on circulating RAGE isoforms in male and female.** cRAGE values were derived by subtracting esRAGE values from the total sRAGE. Bars represent serum levels of total sRAGE, cRAGE and esRAGE in the male and female of three age groups of a healthy population, young (≤45 yrs; male n=17, female n=18-19), middle age (46-64 yrs; male n=34-35, female n=32-37) and elderly-old (≥65 yrs; male n=23, female n=36-37). Values are expressed as median and interquartile range. *P < 0.05; **P < 0.01; ***P < 0.0001; Kruskal-Wallis with Dunn’s for multiple comparison test between age groups of the same gender; Mann Whitney test between male and female of the same age group.

Interactions age*gender revealed non-significant difference with aging in all RAGE isoforms between males and females (sRAGE P= 0.398; esRAGE P=0.380; cRAGE P=0.919). However, sRAGE and cRAGE but not esRAGE, declined differently with gender, in particular females showed a progressive reduction across the age groups, while males evidenced a significant drop already in the middle-age subjects compared to the young ones that remaining unaltered in the elderly-old group (Fig. 2). As consequence, both sRAGE and cRAGE levels of the middle-age females were significantly higher in respect to males (Fig.2).

Thus, our data indicate that among RAGE isoforms, only cRAGE effectively decrease with age and the differences of sRAGE levels associated to aging are influenced by cRAGE variations.

### AGEs and S100A8/A9 but not HMGB1 increase with aging

**Figure 3:**
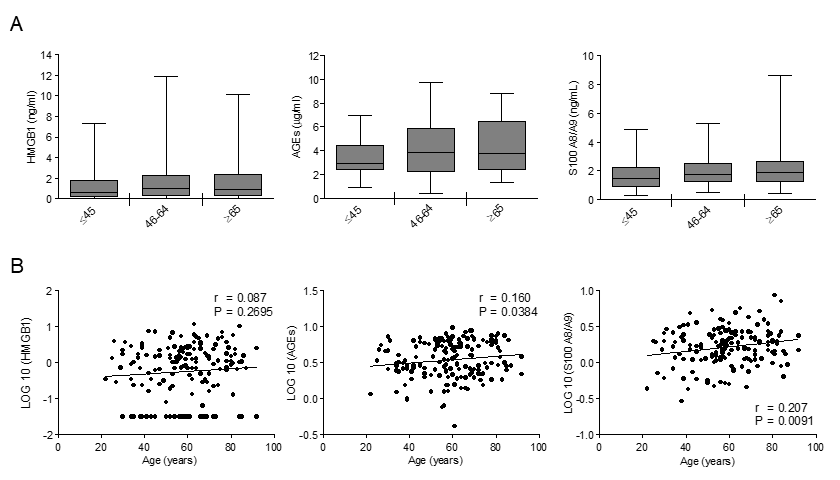
**The effect of age on circulating levels of RAGE ligands: AGEs, S100A8/A9 and HMGB1.** (**A**) Bars represent serum levels of indicated RAGE ligands in three age groups of a healthy population, young (≤45 yrs; n=36), middle age (46-64 yrs; n=65-71) and elderly-old (≥65 yrs; n=57-61). Data are in the original units. Values are expressed as median and interquartile range. Kruskal-Wallis with Dunn’s multiple comparison test. (**B**) Scatter plots showing correlation between age and HMGB1, AGEs or S100A8/A9. Variables showed a skew-ness distribution and were log-transformed. r=Pearson’s coefficient. P < 0.05 was considered statistically significant; n=159-169.

To assess RAGE ligands variations with age, we measured HMGB1, AGEs and S100A8/A9 in our population. HMGB1 did not vary with age (Fig. 3). Both AGEs and S100A8/A9 showed a tendency to increase across the age groups (Fig. 3A) and their levels exhibited a significant positive correlation with age, S100A8/A9 showing the highest correlation coefficient value (Fig. 3B).

Distributions of RAGE ligands were not different between subjects affected and not affected by hypertension, therefore, hypertensive individuals were not removed from the studied population (log_HMGB1 P=0.786; log_S100A8/9 P=0.927; log_AGEs P= 0.993).

### Determinants of serum levels of RAGE isoforms

In order to assess whether in addition to age RAGE isoforms were associated with other variables of our population, we performed a univariate correlation analysis. We found that sRAGE inversely correlates with WC, W/H, insulin and HOMA index, Cholesterol, Ferritin, PAI-1, AGEs, S100A8/A8 and HMGB1; esRAGE inversely correlates with WC, W/H, HGB, insulin and HOMA index, ALT and ferritin, while positively with AGEs; cRAGE inversely correlates with WC, W/H ratio, PAI-1, HMGB1, AGEs, S100A8/A9 and antihypertensive drugs (Table 2). As expected, both esRAGE and cRAGE had high positive correlation with sRAGE (Table 2).

**Table 2:** Univariate correlation analysis between RAGE isoforms and population variables.

	**log sRAGE**			**log esRAGE**		**log cRAGE**	
	**r**	**P**		**r**	**P**	**r**	**P**
**Sex (n/% male)**	-0.118		0.1276	-0.137	0.0856	-0.025	0.7543
**BMI (Kg/m^2^)**	-0.089		0.2514	-0.147	0.0654	-0.027	0.7417
**WC (cm)**	-0.262		**0.0006**	-0.247	**0.0018**	-0.168	**0.0351**
**H (cm)**	-0.020		0.8007	-0.058	0.4710	0.023	0.7791
**W/H**	-0.347		**<0.0001**	-0.290	**0.0002**	-0.256	**0.0012**
**WBC (10^3^/uL)**	-0.026		0.7421	-0.044	0.5864	0.033	0.6792
**RBC (10^6^/uL)**	-0.063		0.4138	-0.149	0.0613	0.012	0.8823
**log Triglycerides (mg/dL)**	0.007		0.9243	-0.025	0.7586	-0.035	0.6614
**Cholesterol (mg/dL)**	0.157		**0.0419**	0.113	0.1584	0.033	0.6827
**HDL (mg/dL)**	0.115		0.1364	0.105	0.1890	0.062	0.4377
**LDL (mg/dL)**	-0.034		0.6581	-0.105	0.1898	-0.062	0.4374
**ApoAI (mg/dL)**	-0.070		0.3689	-0.009	0.9098	-0.065	0.4175
**ApoB (mg/dL)**	-0.007		0.9270	-0.070	0.3861	-0.018	0.8245
**log Insulin (uiU/mL)**	-0.175		**0.0236**	-0.192	**0.0155**	-0.060	0.4509
**Glucose (mg/dL)**	-0.019		0.8093	-0.026	0.7420	-0.006	0.9437
**log HOMA Index**	-0.170		**0.0276**	-0.190	**0.0166**	-0.056	0.4832
**HGB (g/dL)**	-0.089		0.2490	-0.175	**0.0282**	0.017	0.8307
**HbA1c (%)**	-0.004		0.9568	-0.042	0.6031	0.028	0.7268
**GGT (U/L)**	-0.080		0.3033	-0.081	0.3152	-0.108	0.1776
**log AST (U/L)**	-0.044		0.5698	-0.080	0.3181	-0.083	0.3014
**log ALT (U/L)**	-0.060		0.4421	-0.211	**0.0079**	0.075	0.3487
**Transferrin (mg/dL)**	-0.030		0.7013	-0.112	0.1625	0.001	0.9890
**log Ferritin (ng/mL)**	-0.201		**0.0093**	-0.212	**0.0076**	-0.044	0.5876
**log Azotemia (mg/dL)**	-0.100		0.1979	0.011	0.8919	-0.138	0.0843
**Creatinine (mg/dL)**	0.071		0.3635	0.037	0.6443	0.014	0.8654
**log PAI-1 (ng/mL)**	-0.205		**0.0078**	-0.128	0.1090	-0.183	**0.0213**
**log CRP (mg/L)**	-0.113		0.1477	-0.108	0.1791	-0.137	0.0899
**log IL6 (pg/mL)**	-0.114		0.1473	-0.081	0.3236	-0.077	0.3450
**log HMGB1 (ng/mL)**	-0.271		**0.0005**	-0.003	0.9672	-0.290	**0.0002**
**log S100A8/A9 (ng/mL)**	-0.265		**0.0008**	-0.123	0.1242	-0.223	**0.0049**
**log AGEs (μg/mL)**	-0.184		**0.0172**	0.204	**0.0103**	-0.374	**<0.0001**
**log sRAGE (pg/mL)**	-		-	0.600	**0.0001**	0.720	**<0.0001**
**Smoking (n/%)**	0.087		0.2642	0.109	0.1723	0.065	0.4187
**Ipertension (n/%)**	-0.040		0.6047	0.036	0.6502	-0.124	0.1202
**Antihypertensive (n/%)**	-0.011		0.8867	0.124	0.1201	-0.159	**0.0465**
**Antiplatelet (n/%)**	-0.125		0.1077	-0.060	0.4551	-0.116	0.1453
**Gastro protectant (n/%)**	-0.012		0.8806	0.047	0.5608	-0.067	0.4058
**Micronutrients (n/%)**	-0.014		0.8562	0.120	0.1315	-0.111	0.1646
**CNS depressants (n/%)**	0.028		0.7206	0.121	0.1306	-0.144	0.0720
**Thyroid therapy (n/%)**	-0.050		0.5181	-0.059	0.4601	-0.075	0.3508
**Lipid-lowering (n/%)**	-0.039		0.6190	0.004	0.9636	-0.066	0.4101

Variables with Gaussian distribution are expressed as mean ± SD; variables with skewed distribution as median and interquartile range. In bold statistically significant P trend value. r= Pearson correlation coefficient. n=158-169. BMI=body mass index; WC=Waist Circumference; H=Hip; W/H=Waist/Hip ratio; WBC=White blood cells, RBC=Red blood cells; HDL=High Density Lipoprotein; LDL=Low Density Lipoprotein; ApoAI=Apolipoprotein AI; ApoB=Apolipoprotein B; HGB=Hemoglobin; HbA1c=glycated hemoglobin; GGT=Gamma Glutamyl transferase; AST=Aspartate transaminase; ALT=Alanine transferase; PAI‐1=Plasminogen activator inhibitor‐1; CRP=C-reactive Protein; IL6=Interleukin 6; HMGB1=High Mobility Group Box 1; S100A8/A9=Calgranulin A/Calgranulin B; AGEs=Advanced glycation end-products; sRAGE=soluble Receptor for advanced glycation end-products; CNS=Central Nervous System.

To identify the determinants for RAGE isoforms changes in our cohort, we executed a multivariate analysis taking in consideration traditional risk factors, RAGE ligands and anti-hypertensive drugs. sRAGE negatively associated with age, W/H and HMGB1, and positively with Triglycerides; esRAGE inversely associated with W/H and positively with AGEs; cRAGE inversely associated with age, W/H, ferritin, S100A8/A9, HMGB1 and AGEs (Table 3). S100A8/A9 was excluded as a variable from the analysis since its strong correlation with HMGB1 generates instability in the estimates of the multivariable model parameters. However, S100A8/A9 associated with sRAGE (β=-0.2003, SE=0.0927, P=0.0326) and cRAGE (β=-0.6551, SE=0.2730, P=0.0178) but not with esRAGE (β=-0.0514, SE=0.0852, P=0.5473) when it replaced HMGB1 in the same multivariable model.

**Table 3:** Multivariable analysis between RAGE isoforms and population variables.

	**log sRAGE**				**log esRAGE**			**log cRAGE**			
**Variable**	**β**	**SE**	**P**	**β**		**SE**	**P**		**β**	**SE**	**P**
**Age (yrs)**	-0.0062	0.0019	**0.0014**	-0.0017		0.0018	0.3719		-0.0148	0.0043	**0.0008**
**Sex (n/%)**	-0.0395	0.0576	0.4945	-0.0361		0.0546	0.5097		0.0490	0.1282	0.7030
**BMI (Kg/m^2^)**	0.0003	0.0061	0.9623	-0.0040		0.0057	0.4896		0.0075	0.0134	0.5781
**W/H**	-1.0193	0.3778	**0.0079**	-0.9193		0.3643	**0.0128**		-1.8048	0.8557	**0.0368**
**log Triglycerides (mg/dL)**	0.2954	0.1127	**0.0097**	0.2008		0.1034	0.0543		0.2307	0.2429	0.3440
**HDL (mg/dL)**	0.0001	0.0019	0.9803	0.0001		0.0017	0.9671		-0.0003	0.0040	0.9321
**LDL (mg/dL)**	-0.0001	0.0006	0.9142	-0.0002		0.0006	0.6989		-0.0011	0.0014	0.4464
**log Insulin (uiU/mL)**	-0.1818	0.0977	0.0651	-0.1788		0.0915	0.0529		0.0223	0.2150	0.9174
**Glucose (*mg/dL)***	0.0016	0.0023	0.4920	0.0002		0.0021	0.9083		0.0046	0.0050	0.3586
**log Ferritin (ng/mL)**	0.0204	0.0626	0.7451	-0.0641		0.0579	0.2700		0.2980	0.1359	**0.0301**
**log CRP (mg/L)**	0.0102	0.0476	0.8307	-0.0088		0.0436	0.8403		-0.0483	0.1024	0.6384
**log IL6 (pg/mL)**	0.0053	0.0825	0.9490	-0.0366		0.0763	0.6325		0.3081	0.1792	0.0879
**log HMGB1 (ng/mL)**	-0.0641	0.0294	**0.0306**	0.0110		0.0271	0.6843		-0.1865	0.0636	**0.0040**
**log AGEs (μg/mL)**	-0.0588	0.0881	0.5059	0.3219		0.0832	**0.0002**		-0.6662	0.1954	**0.0009**
**Antihypertensive (n/%)**	0.0667	0.0536	0.2160	0.0821		0.0506	0.1072		-0.0010	0.1189	0.9934

In bold statistically significant P value. n=158-169. BMI=body mass index; W/H=Waist/Hip ratio; HDL=High Density Lipoprotein; LDL=Low Density Lipoprotein; CRP=C-reactive Protein; IL6=Interleukin 6; HMGB1=High Mobility Group Box 1; AGEs=Advanced glycation end-products.

Thus, in a general population, among sRAGE isoforms, age is a significant determinant only for cRAGE while W/H ratio is a determinant for both isoforms. cRAGE mainly correlated with inflammation markers while esRAGE with obesity-related and insulin resistance markers.

### AGEs enhance cellular amount of FL-RAGE and reduce cRAGE levels

**Figure 4:**
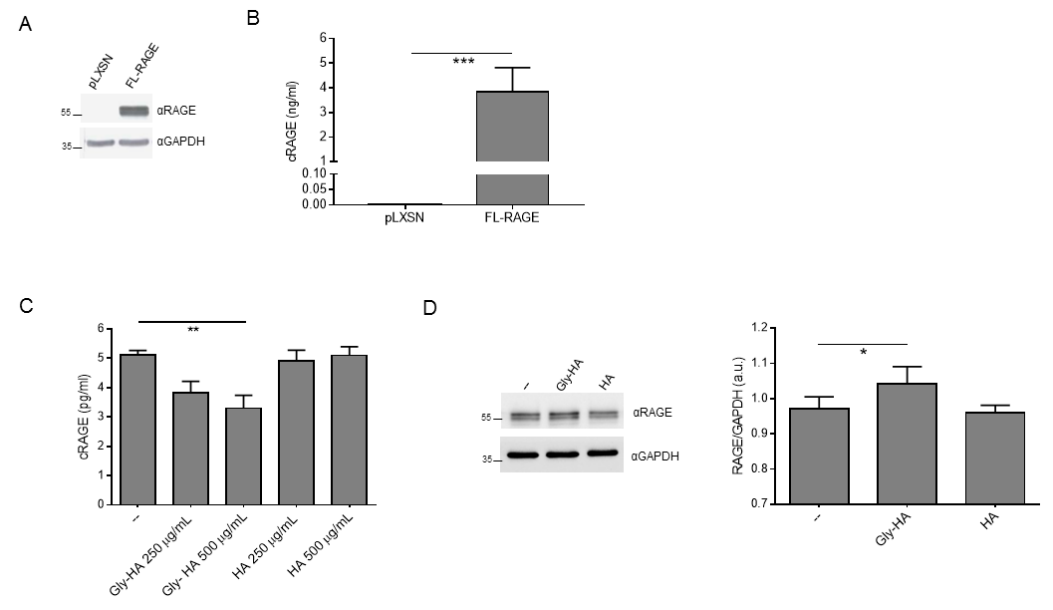
**Effect of glycated albumin on FL-RAGE proteolysis *in vitro.*** (**A**) Expression of FL-RAGE in R3/1-pLXSN (pLXSN) or R3/1-FL-RAGE (FL-RAGE) was determined using 20 µg of protein lysate with a specific antibody raised against the extracellular domain of RAGE. GAPDH detection was used as normalizer. (**B**) Quantification of cRAGE in the supernatant of R3/1-pLXSN (pLXSN) or R3/1-FL-RAGE (FL-RAGE) cells 6 h after medium changing by means of ELISA assay. Data are expressed as mean ± SD. ***, P≤0.0001. Unpaired t test ; n=4. (**C**) Quantification of cRAGE in the supernatant of R3/1-FL-RAGE cells treated or not (--) with indicated concentrations of control human albumin (HA) or glycated human albumin (Gly-HA) for 6 h by means of ELISA assay. Data are expressed as mean ± SD. **, P≤0.01. One-way ANOVA with Bonferroni post-hoc test; n = 4. (**D**) Representative Western blot (left panel) and corresponding quantification (right panel) of FL-RAGE expression using 5 μg protein lysates of R3/1-FL-RAGE cells treated or not (--) with 500 μg/mL HA or Gly-HA for 6 h. GAPDH detection was used as normalizer. Data are expressed as mean ± SD. *, P≤0.05. One-way ANOVA with Bonferroni post-hoc test; n=4; a.u., arbitrary units.

Then, we ought to determine whether RAGE ligands affect cRAGE production and FL-RAGE expression *in vitro*. We generated R3/1 cells, which do not express detectable amount of any endogenous RAGE isoforms, stably expressing FL-RAGE (R3/1/FL-RAGE) or the empty vector (R3/1-pLXSN) (Fig. 4A [2];). FL-RAGE proteolysis is constitutive [24] and indeed, R3/1/FL-RAGE, but not R3/1-pLXSN cells, released in the supernatant a robust amount of cRAGE (Fig. 4B). Since in our cohort AGEs showed the strongest association with cRAGE (Tables 2, 3), we stimulated R3/1/FL-RAGE cells with glycated human albumin (Gly-HA) or control human albumin (HA) and found that the basal production of cRAGE was progressively reduced by increasing concentration of Gly-HA but not by corresponding concentrations of HA (Fig. 4C). Then, we checked expression of FL-RAGE in cell lysates and found that Gly-HA but not HA induced a slight but significant upregulation of FL-RAGE levels.

Thus, these data confirm that AGEs are able to inhibit directly FL-RAGE shedding leading to both membrane-bound FL-RAGE increase and soluble cRAGE reduction.

## DISCUSSION

In the present study, we analysed for the first time the circulating levels of different isoforms of soluble RAGE, i.e. cRAGE and esRAGE, in a cohort of healthy subjects aged 20-90 years-old, to investigate their age-related trends. We show that esRAGE, deriving from alternative splicing [22], does not vary during aging and eventually represents the predominant circulating sRAGE isoform in the elderly-old subjects (Fig. 1B). On the other hand, cRAGE, generated by proteolytic cleavage of FL-RAGE by ADAM10 and/or MMPs [24,26], is the most abundant circulating sRAGE in the young group and significantly decreases with advancing age dropping to very low if any levels in the elderly-old cohort (Fig. 1C). Therefore, the age-related decline of sRAGE in the blood is due to cRAGE changes (Fig.1). No significant gender differences were observed for both isoforms during aging, except for a significant higher concentration of sRAGE in the middle-age group of females compared to males ascribable to cRAGE variations (Fig. 3). Our data extend previously published data showing that in a general population sRAGE levels is slightly but significantly higher in middle-age women compared to men [38,39]. The reason for this is currently unknown, but estrogen levels may be involved [43,44].

Modulation of RAGE isoforms expression in different organs and tissues during healthy aging has not been reported. In physiological conditions, RAGE is predominately expressed in alveolar type I cells of the lung and barely detectable in other cell types [8,10,11,45]. Indeed, human pulmonary lysates presents three RAGE variants (FL-RAGE, cRAGE and esRAGE) and cRAGE is the most abundant one, indicating a continuous and massive shedding of FL-RAGE, while esRAGE levels are low [24,46]. Therefore, it is plausible that the lung contributes as the major source of circulating cRAGE, whose decrease may depend on loss of airway epithelium and/or to a lower expression or activity of ADAM10 or MMPs with aging. Nevertheless, we cannot exclude that inhibition of FL-RAGE proteolysis on other tissues may contribute to age-mediated circulating cRAGE changes. Additional studies in human and experimental models are necessary to clarify this issue.

Importantly, in addition to cRAGE decline, the inhibition of FL-RAGE proteolysis also leads to an increase of its own expression [8]. Notably, our study also reveals that serum levels of RAGE ligands such as AGEs and S100A8/A9 significantly increase with age in healthy subjects (Fig. 3), and HMGB1, AGEs and S100A8/A9 inversely correlate and are independent predictors of cRAGE (Table 2-3). Interestingly, AGEs show the strongest association with cRAGE (Table 2-3), and we demonstrated that these ligands can regulate directly FL-RAGE/cRAGE ratio, since *in vitro* stimulation of alveolar-like cells with glycated albumin reduces the constitutive production of cRAGE and enhances FL-RAGE protein expression (Fig 4). Notably, we also reported that in our population circulating AGEs are positive independent predictors of esRAGE (Table 3) indicating a possible feedback mechanism to counterbalance cRAGE downregulation.

It is well established that, the binding of FL-RAGE with ligands drives a cascade of signaling that converges at nuclear factor-κB (NF-κB) promoting the secretion of pro-inflammatory molecules, recruitment of inflammatory cells to damaged tissue and cell adhesion [3–7,47]. Therefore, the incremental FL-RAGE/ligands axis activation during aging could contribute to the chronic, systemic and low-grade inflammatory condition, named “inflammaging”, typical of elderly, and to the development of age-related diseases, such as diabetes, atherosclerosis and cancer [48]. In these contexts, AGEs have been extensively shown to play a pivotal role [49].

Additionally, since both isoforms of sRAGE can act as a decoy receptor suppressing FL-RAGE/ligands pro-inflammatory and chemotactic activity [8,14,24,27], the progressive decrease of cRAGE with aging could further free RAGE ligands to fuel the inflammatory signals favoring the onset of age-related pathologies. Accordingly, in our cohort, cRAGE inversely correlates with the inflammatory marker PAI-1 (Table 2), known to increase in age-associated clinical conditions including cardiovascular diseases, type 2 diabetes (T2DM), obesity and inflammation [50] and shows also an almost significant inverse correlation with CRP (Table 2).

Experimental animal models suggest a protective role of sRAGE against inflammation associated with obesity and T2DM [14,28]. Several clinical studies have also reported significant negative correlation of sRAGE or esRAGE with BMI, WC, W/H and other obesity indices in a general population, obese and pre-diabetic subjects [36–39,51–53]. Much less is known about cRAGE variations in these pathological contexts. Our data expand previous published reports demonstrating that in a healthy population, W/H negatively associates with and is an independent predictor of both RAGE isoforms (Table 2-3); interestingly, we also found that esRAGE inversely correlates with insulin, HOMA index and ALT and sRAGE correlations with these insulin resistance-related markers depend exclusively on esRAGE variations (Table 2).

How W/H influences cRAGE and esRAGE generation is not known. Recent evidences describe that weight loss by dietary interventions increases esRAGE [52,54] but not cRAGE levels [54] indicating that only the mechanism governing esRAGE production is directly affected by fat deposition changes and is reversible. Likely, the influence of W/H on cRAGE levels is an indirect consequence of body composition changes, such as weight gain and accumulation of abdominal fat, occurring during aging [55]. Accordingly, increased visceral fat mass is a significant contributor of low-grade systemic inflammation and in our cohort cRAGE inversely correlates with PAI-1 (Table 2) which is largely produced by abdominal adipose tissue [56,57].

A limitation of this study is the small number of enrolled subjects and the cross-sectional design. Furthermore, several polymorphisms of RAGE gene (*AGER*) have been identified, including the SNP Gly82Ser that has been associated to decreased sRAGE serum concentrations [58] and lung function [59]. We did not analysed the influence of this genotype on cRAGE and esRAGE levels in our population. Another limitation is the ELISA assay used for AGEs quantification that does not distinguishes between multiple AGEs species.

In conclusion, our finding indicates that in a healthy population, among sRAGE variants, esRAGE represents a more consistent marker of metabolic perturbations associated to insulin resistance and obesity, while cRAGE is a trustworthy biomarker of physiological aging and inflammation. Hence, both RAGE isoforms may be associated with different age-related diseases and their risk factors and could be innovative parameters for the estimation of “biological age”. Further studies are needed to confirm this hypothesis.

## MATERIALS AND METHODS

### Study population

Participants were recruited from the Italian National Research Center on Aging (INRCA), Ancona. All subjects gave their written informed consent to participate in the study, which was approved by INRCA’s Ethics Committee. Serum of roughly 169 healthy subjects aged 20-90 years (yrs) (M=75, F=94) was tested for sRAGE (n=169), esRAGE (n=161), cRAGE (n=161), HMGB1 (n=164), S100A8/A9 (n=158), AGEs (n=168) and IL6 (n=164) along with other variables (Table 1). The health status of subjects was assessed using standardized questionnaires, laboratory assays and physical examination. Subjects were considered healthy if at the time of blood collection they did not have any major acute and/or chronic age-related disease such as AMI, CHF, Alzheimer’s disease (AD), T2DM or cancer. Subjects with a Cumulative Illness Rating Scale (CIRS) > 2, which indicates a comorbid state, were excluded [60]. All the studied subjects consumed a Mediterranean diet.

Subjects were defined as hypertensive when they were under active treatment or when their systolic blood pressure was >140 mmHg and/or their diastolic blood pressure was >90 mmHg, on three different occasions. Body mass index (BMI) was determined as body weight in Kg over height in m^2^. Waist circumference (WC) is measured at the midpoint between the lower margin of the last palpable ribs and the top of the iliac crest using a measuring tape. Hip circumference (H) is measured around the widest portion of the buttocks. For both measurements, the individual is standing and wearing little clothing. The measurements are taken at the end of a normal respiration. W/H is calculated as waist measurement divided by hip measurement.

### Laboratory assays

Overnight fasting venous blood samples of all subjects were collected from 8:00 to 10:00 a.m in EDTA and citrate tubes. White blood cell, monocyte and platelet counts were performed by standard automated procedures (Sysmex XE-2100, Kobe, Japan). Blood concentrations of glycosylated hemoglobin (HbA1c) were measured by a G8 HPLC analyzer (TOSOH BIOSCIENCE, USA). An immunoenzymatic method was used for PAI-1 antigen (Biopool, Sweden). Total and HDL cholesterol, fasting insulin, fibrinogen, and apolipoprotein AI and B (ApoAI and ApoB), triglycerides, creatinine, and fasting glucose were measured using commercially available kits on an automated clinical chemistry COBAS analyzer (Roche-Hitachi, Basel, Switzerland). Highly sensitive C-reactive protein (hsCRP) was determined by the particle-enhanced immunoturbidimetric assay (CRP High Sensitive, Roche-Hitachi) on a COBAS analyzer. HOMA index was calculated as Glucose (mg/100 ml)* Insulin (uIU/ml)/405.

### RAGE isoforms and ligands measurements

Whole human peripheral blood was collected in a tube without anticoagulants. Once collected, the blood was left to clot undisturbed at room temperature for 15–30 minutes. The clot was removed by centrifuging at 2000 g for 10 minutes in a refrigerated (4°C) centrifuge. The resulting supernatant was immediately transferred into a clean polypropylene tubes, aliquoted and stored at -80°C. ELISA kits were used to test serum levels of S100A8/A9 (EKMRP8/14 Bühlmann Laboratories Ag, Switzerland), HMGB1 (ST51011, IBL International-Tecan, Switzerland), AGEs (STA-518 Cell Biolabs, INC. San Diego, CA) and IL6 (HS600B, R&D Systems Inc., MN, USA) following manufacturer’s instruction. Total human sRAGE was determined by a commercial ELISA (DY1145, Human RAGE DuoSet ELISA, R&D Systems Inc.) and included the detection of both cRAGE and esRAGE variants. Serum esRAGE concentration was detected by an ELISA kit with a specific antibody raised against the unique C-terminal 9 amino acids (332-347) sequence (K1009-1, B-bridge International, CA, USA). Serum cRAGE was determined by subtracting esRAGE from sRAGE as already described [36,54]. cRAGE values equal to zero were imposed to be identical to the lowest determined value.

### Cell culture

Rat alveolar type I-like R3/1 cells and derived clones were grown in Dulbecco modified Eagle medium (DMEM) supplemented with 10% FBS and 1% penicillin/streptomycin. To generate R3/1-pLXSN or R3/1-FL-RAGE clones, R3/1 cells were infected with retrovirus carrying p-LXSNneo or p-LXSN-neo-FL-RAGE retrovectors. Clones were selected with 500 mg/ml G418 ([2]; Invitrogen, Carlsbad, California, USA).

### Assessment of FL-RAGE expression and cRAGE production

Two thousand R3/1-pLXSN or R3/1-FL-RAGE clones were seeded on 6 well-plate (Costar, Kennebunk, ME, USA) and treated with indicated concentration of human glycated Albumin (HGA; #A8301, Sigma Aldrich, Saint Louis, Missouri, USA) or not-glycated human Albumin (HA; #A7736, Sigma Aldrich) as control in DMEM supplemented with 0.1% FBS for 6 hours. Supernatant of cells was tested for cRAGE concentration by a commercial ELISA (DY1145, Human RAGE DuoSet ELISA, R&D Systems Inc., MN, USA). Cells were harvested in RIPA buffer in the presence of proteases inhibitors (P8849, Sigma-Aldrich), incubated in ice for 30 min and centrifuged at 12000g for 15 minutes at 4°C. Protein extracts were separated by SDS-PAGE and transferred to Amersham Hybond ECL Nitrocellulose membranes (GE Healthcare, Little Chalfont, United Kingdom). Membranes were incubated with a goat anti-human RAGE (1 µg/ml; cat. AF1145; R&D Systems Minneapolis, MN, USA) or an antibody against GAPDH (0.2 µg/mL, sc-25778, Santa Cruz Biotechnology) as a loading control. Proteins were visualized by an enhanced chemiluminescence (ECL) detection system (cat. RPN2106, GE Healthcare) and acquired with a ChemiDoc™ MP Imaging System (Biorad, Hercules, CA, USA). Protein bands were quantified by densitometry analysis using ImageJ.

### Statistical analysis

Qualitative variables were reported as frequencies and percentages. Quantitative variables were reported as mean plus standard deviation (continuous variables normally distributed) or median and interquartile range (continuous variables skewed distributed). Skewed distributed variables were transformed in logarithm to the base 10: Triglycerides, Insulin, HOMA Index, AST, ALT, Ferritin, Azotemia, PAI-1, CRP, IL6, HMGB1, S100A8/A9, AGEs, sRAGE, esRAGE and cRAGE. Population features changes were classified according to age into three classes, young (≤45 yrs), middle age (46-64 yrs) and elderly-old (≥65 yrs) and linear trends across categories were evaluated. T-test was used to compare means between two groups. Non-parametric ANOVA Kruskal-Wallis with Dunn’s multiple comparisons test between different age classes by gender or Mann Whitney test between male and female of the same age class were applied. The crude relation between RAGE isoforms and the other variables were assessed by Pearson correlation coefficient for continuous variables and by Spearman rank correlation for dichotomous variables. The independent effect of each variable on RAGE isoforms from the other variables were evaluated by multivariable linear regression analysis. Statistical interactions age*gender were also computed to evaluate different effect of age on RAGE isoforms by gender. *In vitro* experiments were performed at least four times. The Shapiro-Wilk test was used to assess the normality of distribution of investigated parameters. Differences between two groups or more than two groups were conducted with unpaired Student's t-test or one-way analysis of variance (ANOVA) with Bonferroni post-hoc test, respectively, and values were presented as mean ± SD. The analyses were performed using SAS9.4 program or GraphPad Prism software version 7 (GraphPad Software, Inc, La Jolla, CA, USA). A value of p <0.05 was considered statistically significant.

## SUPPLEMENTARY MATERIAL

Supplementary Table 1
